# Estimating the sample size for a pilot randomised trial to minimise the overall trial sample size for the external pilot and main trial for a continuous outcome variable

**DOI:** 10.1177/0962280215588241

**Published:** 2015-06-19

**Authors:** Amy L Whitehead, Steven A Julious, Cindy L Cooper, Michael J Campbell

**Affiliations:** 1 Medical Statistics Group, Design, Trials and Statistics Group, School of Health and Related Research, University of Sheffield, Sheffield, UK; 2 Clinical Trials Research Unit, Design, Trials and Statistics Group, School of Health and Related Research, University of Sheffield, Sheffield, UK

**Keywords:** Pilot trial, RCT, sample size, power, continuous outcome

## Abstract

Sample size justification is an important consideration when planning a clinical trial, not only for the main trial but also for any preliminary pilot trial. When the outcome is a continuous variable, the sample size calculation requires an accurate estimate of the standard deviation of the outcome measure. A pilot trial can be used to get an estimate of the standard deviation, which could then be used to anticipate what may be observed in the main trial. However, an important consideration is that pilot trials often estimate the standard deviation parameter imprecisely. This paper looks at how we can choose an external pilot trial sample size in order to minimise the sample size of the overall clinical trial programme, that is, the pilot and the main trial together. We produce a method of calculating the optimal solution to the required pilot trial sample size when the standardised effect size for the main trial is known. However, as it may not be possible to know the standardised effect size to be used prior to the pilot trial, approximate rules are also presented. For a main trial designed with 90% power and two-sided 5% significance, we recommend pilot trial sample sizes per treatment arm of 75, 25, 15 and 10 for standardised effect sizes that are extra small (≤0.1), small (0.2), medium (0.5) or large (0.8), respectively.

## 1 Introduction

Sample size is an important consideration when a clinical trial is planned, not only for the main trial but also for any preliminary pilot trial. A sample size calculation is used to determine the minimum number of participants needed in a clinical trial in order to be able to answer the research question under investigation.^1^ Recruiting too few participants in a main trial means that the probability of finding a clinically relevant difference statistically significant is low and as a consequence, the chance of inconclusive results is high.^2,3^ Conversely, if the sample size is too large, resources may be wasted, more patients than necessary could be given a treatment which will later be proven to be inferior; or an effective treatment may be delayed from being released on to the market.^4^

For the purpose of this work, we are defining a pilot randomised trial as a trial, which mimics the design of the main trial but is not designed with the aim to prove the superiority of one treatment over another^5^ but rather to try out aspects of the proposed main trial. As pilot trials do not have the same objectives as a main trial, setting the sample size in the same way – using formal power considerations – is usually not necessary. However, it is still necessary to provide a sample size justification even when the reasons for choosing a particular size are pragmatic.

The focus of this paper will be deriving pilot trial sample sizes based on a primary aim of the pilot being to estimate the standard deviation to be used for the main trial sample size calculation. We will describe a method for estimating the sample size for a pilot trial, which achieves the objective of minimising the recruitment of patients across the pilot and the main trial overall. The emphasis in this paper is on two armed superiority trials; however, the results are easily generalisable to trials with other designs. Furthermore, we will concentrate on external pilot trials where the assumption, however, is that there are no changes between it and the main trial, so that the standard deviation of the outcome measurement is unaffected. We are also not considering the situation of an internal pilot trial where the data are combined from the pilot trial and the main trial for the final analysis.

## 2 Standard methods

For a continuous normally distributed outcome, in a superiority trial, the sample size per treatment arm, *n*, to ensure adequate power (1–β) where β is the Type II error rate whilst controlling the Type I error rate, α, for a specified/required treatment difference, *d*, and standard deviation, σ, is given by

(1) $$\frac{n=(r+1)(z_{1-\beta }+z_{1-\alpha /2}{)}^{2}\sigma^{2}}{{\mathrm{rd}}^{2}}$$

where *r* is the allocation ratio of participants between the two treatment arms, experimental to control.^6^

Subjective clinical expertise can be used to specify the required treatment difference and there are agreed values used for the Type I and II error levels. However, a difficulty arises when trying to quantify the standard deviation.^7^ Estimating the standard deviation at an inappropriate level can have a serious effect on the power of the study.^8^ If the anticipated standard deviation is estimated to be too high, the trial will contain more participants than necessary. If the anticipated value is estimated to be too low, the trial will not contain enough participants to find the required effect, leading to the problems outlined in Section 1.

One of the methods investigators might use to try to get an accurate prediction of the true standard deviation (or variance) of the outcome measure is to conduct an external pilot trial prior to the main trial. Pilot trials are often small; therefore, they tend to imprecisely predict the true variance. The anticipated distribution of the pilot variance is a chi-squared distribution.^9^ As a consequence, the accuracy of the variance prediction will depend on the pilot sample size and, hence, the degrees of freedom for the variance. Estimating the main trial sample size from equation (1) can result in a loss of power when the variance is imprecisely estimated. Using previous trial results to estimate the variance introduces a type of imprecision that should be allowed for when estimating the sample size for the main trial.^9^

### 2.1 Adjusting the standard deviation estimate from a pilot trial

Two different methods have been developed to try to deal with the issue of imprecise variance estimates. The first was proposed by Browne^10^ and will be referred to as the upper confidence limit (UCL) approach and the second by Julious and Owen^9^ which will be referred to as the non-central t-distribution (NCT) approach. In both methods, the sample size is inflated to allow for the imprecision involved when estimating the variance of an outcome measure from a pilot trial.

#### 2.1.1 UCL approach

The UCL approach uses an 100*X*% UCL for the estimated value of the variance from the pilot trial to plan the main trial. Browne^10^ contended that this provides a sample size sufficient to achieve the required power in at least 100*X*% of such trials. Browne recommends an 80% upper confidence level. However, Sim and Lewis,^11^ whose results will be discussed later in the paper, set *X* at 0.95 or the 95% level.

In order to implement the UCL approach, a variance estimate from the pilot data is obtained and the one-sided *X*% UCL for this variance, $s_{\mathrm{UCL}}^{2}$, is calculated. A one-sided 100X% UCL for the variance can be calculated from

(2) $$s_{\mathrm{UCL}}^{2}=[\frac{k}{\chi_{1-X,k}^{2}}]s^{2}$$

where *s*^2^ is the pooled variance from the pilot trial with *k* degrees of freedom for the variance estimate, and $\chi_{1-X,k}^{2}$ denotes the 1-X percentile of the chi-squared distribution with *k* degrees of freedom.^12^ As *k* increases, the confidence interval for a variance estimate becomes narrower.

Note for a two arm parallel group pilot trial with equal allocation to treatments, *k* would usually be k=2m-2, where *m* is the sample size per arm in the pilot trial from which the variance is being estimated.

This UCL would, therefore, be used as the variance estimate in the traditional sample size equation given earlier in equation (1). Therefore, the sample size per treatment arm for the main trial, *n_M_*, would be given by

(3) $$n_{M}=\frac{(r+1)(z_{1-\beta }+z_{1-\alpha /2}{)}^{2}s_{\mathrm{UCL}}^{2}}{{\mathrm{rd}}^{2}}.$$

If we investigate how much larger the sample size estimate is from this approach compared to the standard approach, by dividing equation (3) by equation (1) with *s*^2^ used as an estimate of $\sigma^{2}$, we find that the UCL approach sample size is larger by a factor of $\left[\frac{k}{\chi_{1-X,k}^{2}}\right]$. Therefore, the factor by which the UCL approach sample size is greater than the standard approach depends only upon the pilot trial sample size and the value of *X*. It is possible, therefore, to calculate inflation factors, which can be used to multiply by the sample size from a standard calculation to give the sample size for the UCL approach for a set value of total pilot trial sample size and *X*; these can be seen in Table 1. The pilot trial sample sizes used here are total sample sizes across treatment arms – assuming for the purpose of this paper, the trial is a two armed trial.

**Table 1.** Inflation factors for the sample size calculation using the UCL approach.

Pilot trial sample size	80% upper confidence limit	95% upper confidence limit
20	1.400	1.917
24	1.349	1.783
30	1.297	1.654
40	1.244	1.527
50	1.211	1.450
70	1.172	1.359
100	1.139	1.287
200	1.093	1.190

#### 2.1.2 NCT approach

Julious and Owen^9^ suggest an alternative method for the calculation of sample size accounting for the fact that we are using a sample estimate of the variance rather than the population variance in the calculation. The sample size inflation is dependent on the number of degrees of freedom on which the estimate of the variance is based, *k*; therefore, the sample size per treatment arm for the main trial, *n_M_*, would be given by

(4) $$n_{M}\geq \frac{(r+1)[t^{-1}(1-\beta ,k,t^{-1}(1-\alpha /2,n_{M}(r+1)-2,0)){]}^{2}s^{2}}{{\mathrm{rd}}^{2}}$$

where $t^{-1}(.,k,a)$ is the inverse function of the cumulative distribution function of a NCT with a non-centrality parameter, *a*, on *k* degrees of freedom. The non-centrality parameter in this case is $t^{-1}(1-\alpha /2,n_{M}(r+1)-2,0)$ which is the inverse function of the cumulative distribution function of a central t-distribution with $n_{M}(r+1)-2$ degrees of freedom (as a = 0). Here *k* is the degrees of freedom for the variance estimate *s*^2^. If the estimate of the variance is based on only a few degrees of freedom, the sample size will be increased. Consequently, as the number of degrees of freedom for the estimate of the variance increases, the impact of this method on the sample size diminishes. As can be seen in the paper by Julious and Owen,^9^ it is also possible to calculate inflation factors for the NCT approach. The inflation factor represents how much larger the NCT approach sample size would be compared to the standard sample size calculation. Table 2 shows the inflation factors for this approach for total pilot trial sample sizes.

**Table 2.** Inflation factors for the sample size calculation for the NCT approach when the Type I error is 5%.

Pilot trial sample size	Power	
	90%	80%
20	1.156	1.099
24	1.125	1.080
30	1.097	1.062
40	1.071	1.045
50	1.055	1.036
70	1.039	1.025
100	1.027	1.017
200	1.013	1.009

The UCL approach inflation depends only on the pilot trial sample size and the chosen level of *X*, whereas the NCT inflation factor depends on the pilot trial sample size and the Type I and Type II error rates. We can see from Tables 1 and 2 that the inflation factors for the UCL approach when *X* is 80 or 95% are much higher than the inflation factors for the NCT approach. Table 3 demonstrates which value of *X* in the UCL approach would make the inflation factor equal to that of the NCT method, as well as the resulting inflation factor, the sample sizes presented are total pilot trial sample sizes.

**Table 3.** Inflation factors and levels of *X* for the UCL approach that give the same sample size as the NCT approach.

Pilot trial sample size	Power			
	90%		80%	
	*X*	Inflation factor	*X*	Inflation factor
20	0.622	1.156	0.566	1.099
24	0.611	1.125	0.560	1.080
30	0.599	1.097	0.553	1.062
40	0.586	1.071	0.546	1.045
50	0.577	1.056	0.541	1.036
70	0.565	1.039	0.534	1.025
100	0.554	1.027	0.529	1.017
200	0.538	1.013	0.520	1.008

It can be seen that as the pilot sample size increases the value for *X* in the UCL approach, which would lead to the same sample size as the NCT approach tends towards 0.5 and the inflation factor tends towards 1.

### 2.2 Pilot trial sample sizes

So far, we have highlighted how to estimate the sample size for a main trial based on the estimates of variance from a pilot trial. The question now being considered is how to estimate the sample size for the pilot trial in the situation where the variance estimate from the pilot trial is being used to design a main trial.

As highlighted previously, in a pilot trial the objective is not to prove superiority of the treatment but to test trial procedures and processes and to get estimates of parameters for the main trial sample size calculation.^13–15^ Therefore, the sample size formulae which are used for main treatment assessments are not usually applicable to pilot trials. The Consolidated Standards of Reporting Trials Group and bodies such as The National Institute for Health Research and The National Research Ethics Service state that not all studies necessarily need a power-based sample size calculation but they do all need a sample size justification. Therefore, since the purpose of the pilot is not to give a formal assessment of efficacy, then the sample size provided by the conventional calculations may be higher than necessary.^13^

#### 2.2.1 Rules of thumb

When estimating the sample size for the pilot trial, the simplest methods to apply are sample size rules of thumb. Browne^10^ cites a general flat rule to ‘use at least 30 subjects or greater to estimate a parameter’, whereas Julious^16^ suggests a minimum sample size of 12 subjects per treatment arm. Teare et al.^17^ recommend a pilot trial sample size of 70 in order to reduce the imprecision around the estimate of the standard deviation. All of these rules have limitations, however, as they are applied regardless of the size of the main trial being designed. The cost of the simplicity of this flat approach, is a larger overall sample size when the main trial is large or small, as demonstrated in Section 4.

#### 2.2.2 Minimising the sample size across studies approach

If one of the adjustment methods described in the previous section to account for imprecision in the variance estimation is applied to calculate the main trial sample size, it would mean that the pilot trial sample size would affect the sample size of the main trial. That is, the methods depend on the degrees of freedom around the variance estimate and hence the pilot sample size.

There is a trade-off, therefore, between having a small pilot study and a larger main trial or a larger pilot study and a smaller main trial. This is because the larger the pilot the more precisely estimated the variance will be and, hence, the smaller the inflation factor applied to the main study sample size calculation. However, eventually the pilot sample size will get too large, and the number included in the pilot trial will outweigh the reduction in the main trial sample size. Therefore, it may be appropriate to consider the implications of this relationship when choosing the sample size of the pilot trial.

The method of setting the pilot trial sample size in order to minimise the overall sample size of the pilot and the main trial together was described by Kieser and Wassmer.^12^ They applied the 80% UCL approach to the sample size calculation and found that a pilot trial sample size between 20 and 40 would minimise the overall sample size for a main study sample size of 80–250 corresponding to standardised effect sizes of 0.4 and 0.7 (for 90% power based on a standard sample size calculation). Sim and Lewis^11^ also applied the UCL approach in their work but with a 95% UCL. They found that a pilot trial of *n* ≥ 55 would minimise the overall sample size for small to medium standardised effect sizes (0.2–0.6). The impact of Sim and Lewis’ use of a 95% UCL is that it has the effect of increasing their estimate of the required sample size compared to Kieser and Wassmer, for both the pilot and the main trial.

The current methods for setting pilot trial sample sizes are based on a set of rules, which we will call flat rules of thumb, these are given in Table 4. These pilot sample sizes are fixed no matter how large the subsequent main trial will be.

**Table 4.** The current flat rules of thumb for overall pilot trial sample size of a two armed trial.

Author	Recommended pilot trial sample size
Julious^16^	24
Kieser and Wassmer^12^	20–40
Browne^10^	30
Sim and Lewis^11^	≥55
Teare et al.^17^	70

**Figure 1.**
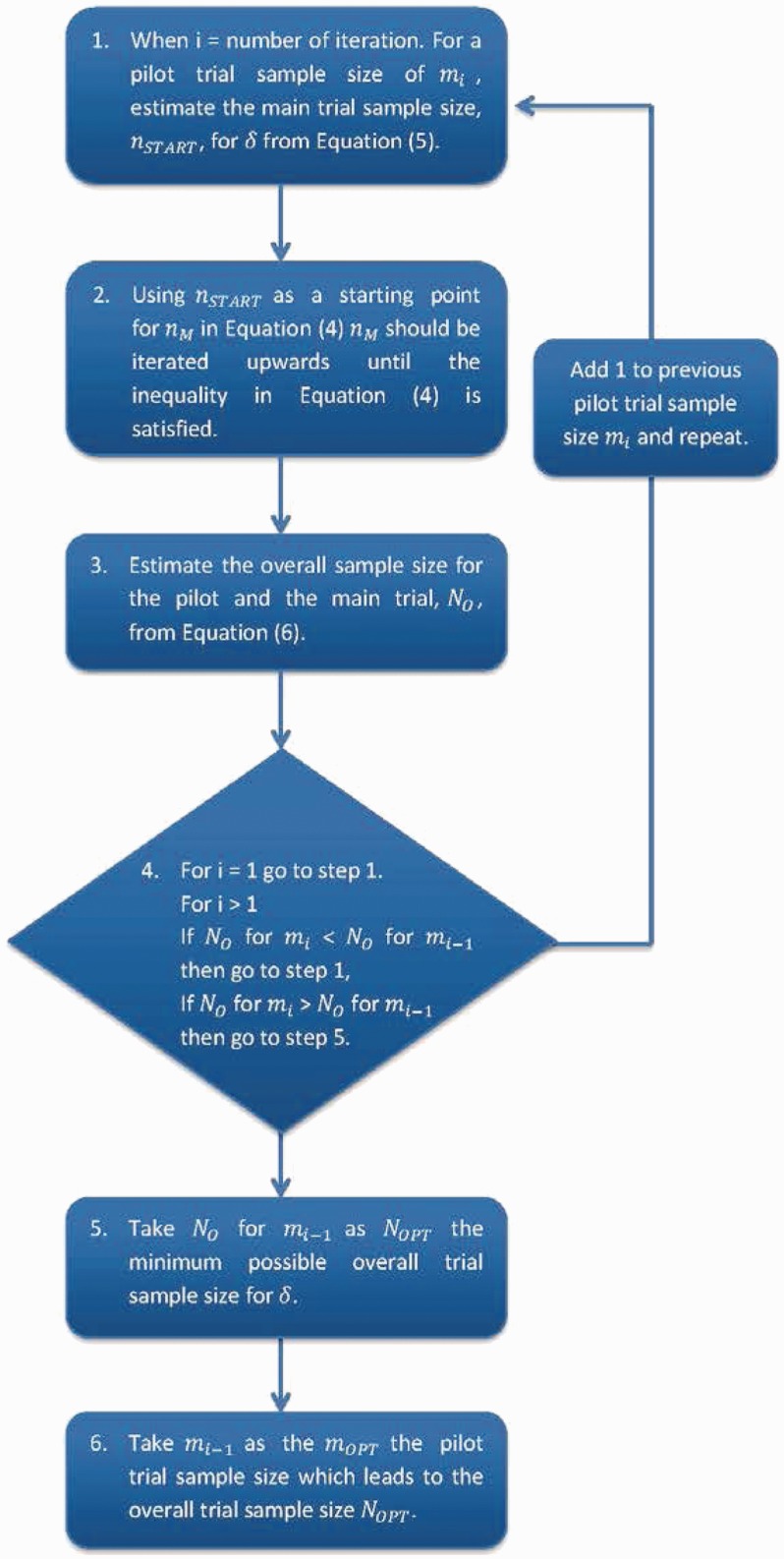
Process for calculating the optimal pilot trial sample size.

**Figure 2.**
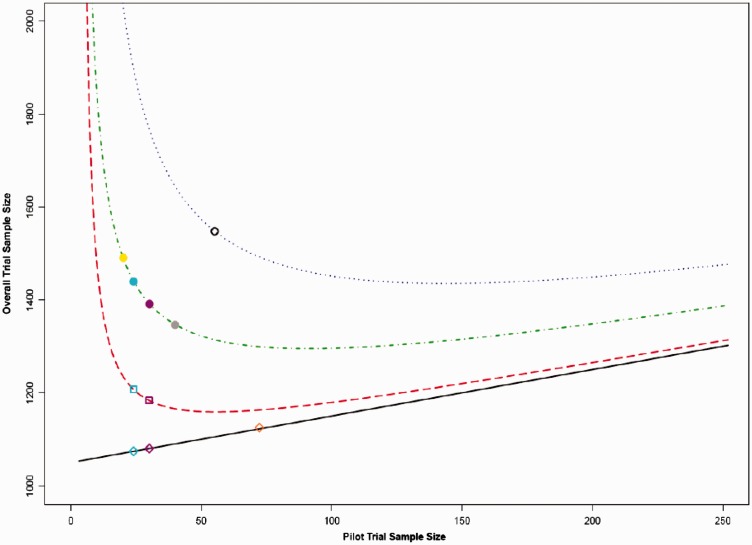
Comparing overall total trial sample sizes for each adjustment method over varying pilot trial sample size for a standardised difference of 0.2. *Lines from bottom to top: Line 1, Standard sample size calculation with no adjustment method applied (points represent pilot trial sample sizes of 24, 30 and 70); Line 2, Main trial sample size calculation based on the NCT approach (points represent pilot trial sample sizes of 24 and 30); Line 3, Main trial sample size calculation based on the 80% UCL approach (points represent pilot trial sample sizes of 20, 24, 30 and 40) and Line 4, Main trial sample size calculation based on the 95% UCL approach (point represents pilot trial sample size of 55).

**Figure 3.**
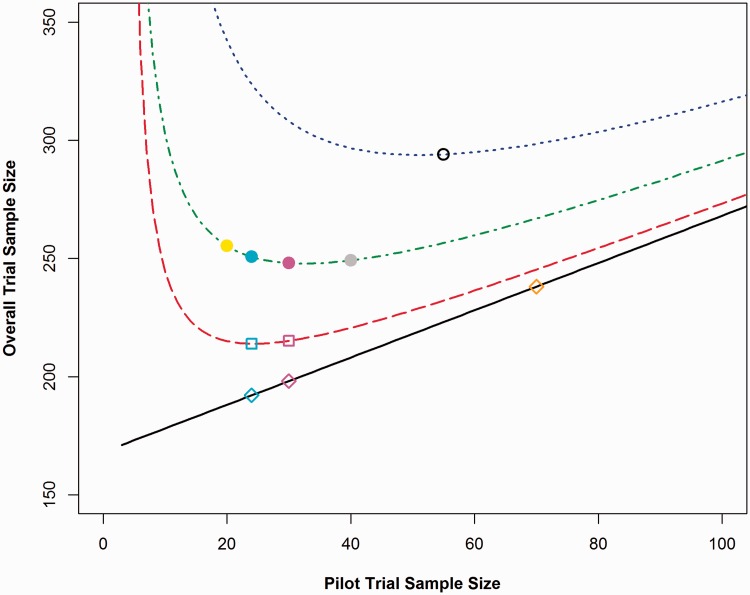
Comparing overall trial sample sizes for each adjustment method for varying pilot trial sample sizes for a standardised difference of 0.5. *Lines from bottom to top: Line 1, Standard sample size calculation with no adjustment method applied (points represent pilot trial sample sizes of 24, 30 and 70); Line 2, Main trial sample size calculation based on the NCT approach (points represent pilot trial sample sizes of 24 and 30); Line 3, Main trial sample size calculation based on the 80% UCL approach (points represent pilot trial sample sizes of 20, 24, 30 and 40) and Line 4, Main trial sample size calculation based on the 95% UCL approach (point represents pilot trial sample size of 55).

**Figure 4.**
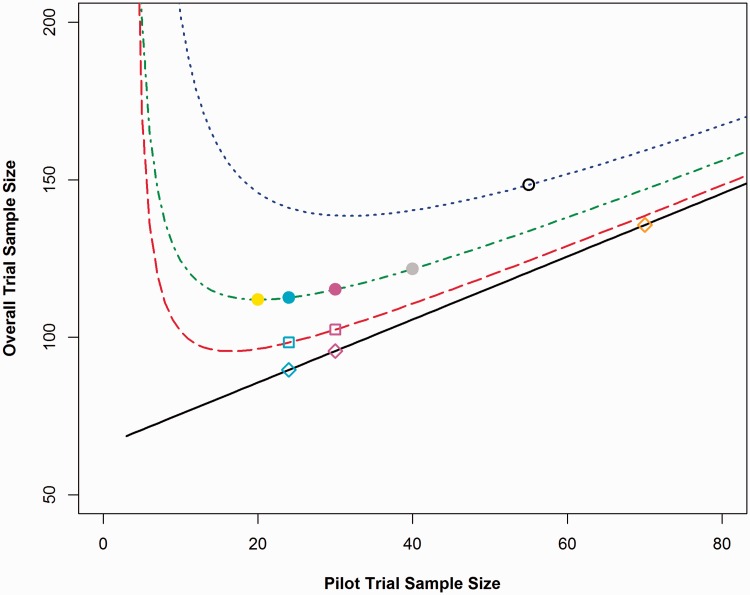
Comparing overall trial sample sizes for each adjustment method for varying pilot trial sample sizes for a standardised difference of 0.8. *Lines from bottom to top: Line 1, Standard sample size calculation with no adjustment method applied (points represent pilot trial sample sizes of 24, 30 and 70); Line 2, Main trial sample size calculation based on the NCT approach (points represent pilot trial sample sizes of 24 and 30); Line 3, Main trial sample size calculation based on the 80% UCL approach (points represent pilot trial sample sizes of 20, 24, 30 and 40) and Line 4, Main trial sample size calculation based on the 95% UCL approach (point represents pilot trial sample size of 55).

Please note that the sample sizes presented in Tables 1 to 4 and in Figures 2 to 4 are the total sample size required for a two arm trial. This has been done to allow for comparisons to be made between the flat rules of thumb: as some rules are based on the numbers of participants required per arm and some are based on the total number of participants required – for example, Sim and Lewis^11^ recommend 55 or more patients in total. The results presented in Figure 1 and Tables 5, 6 and 8 are per treatment arm. This allows for generalisability to trials with two or more treatment arms.

**Table 5.** Theoretical optimal values of pilot trial, main trial and overall trial sample size per treatment arm for each inflation method.

	Inflation method								
Standardised difference	80% upper confidence limit			95% upper confidence limit			Non-central t-distribution		
	Pilot	Main	Overall	Pilot	Main	Overall	Pilot	Main	Overall
80% powered main trial									
0.05	210	6671	6881	331	6892	7223	74	6353	6427
0.10	88	1728	1816	139	1817	1956	38	1607	1645
0.20	39	457	496	61	493	554	20	412	432
0.25	30	300	330	47	326	373	16	267	283
0.30	24	213	237	38	234	272	14	188	202
0.40	18	125	143	28	139	167	11	108	119
0.50	14	83	97	22	94	116	9	71	80
0.60	12	60	72	18	69	87	8	51	59
0.70	10	45	55	16	53	69	7	38	45
0.75	10	40	50	15	47	62	7	33	40
0.80	9	36	45	14	42	56	6	30	36
0.90	8	29	37	13	35	48	6	24	30
1.00	7	25	32	11	29	40	5	20	25
90% powered main trial									
0.05	253	8880	9133	398	9149	9547	106	8511	8617
0.10	106	2292	2398	167	2400	2567	54	2154	2208
0.20	46	603	649	72	647	719	28	552	580
0.25	35	394	429	56	427	483	23	358	381
0.30	29	279	308	45	305	350	19	252	271
0.40	21	163	184	33	181	214	15	145	160
0.50	16	108	124	26	122	148	12	95	107
0.60	14	78	92	21	89	110	11	68	79
0.70	12	59	71	18	68	86	9	51	60
0.75	11	52	63	17	60	77	9	45	54
0.80	10	46	56	16	54	70	8	40	48
0.90	9	38	47	14	44	58	8	32	40
1.00	8	32	40	13	37	50	7	27	34

**Table 6.** Theoretical optimal values of pilot trial, main trial and overall trial sample size per treatment arm for each inflation method with a floor on the lower limit of pilot trial sample size at 10 per arm.

	Inflation method								
Standardised difference	80% upper confidence limit			95% upper confidence limit			Non-central t-distribution		
	Pilot	Main	Overall	Pilot	Main	Overall	Pilot	Main	Overall
80% powered main trial									
0.05	210	6671	6881	331	6892	7223	74	6353	6427
0.10	88	1728	1816	139	1817	1956	38	1607	1645
0.20	39	457	496	61	493	554	20	412	432
0.25	30	300	330	47	326	373	16	267	283
0.30	24	213	237	38	234	272	14	188	202
0.40	18	125	143	28	139	167	11	108	119
0.50	14	83	97	22	94	116	10	70	80
0.60	12	60	72	18	69	87	10	49	59
0.70	10	45	55	16	53	69	10	36	46
0.75	10	40	50	15	47	62	10	31	41
0.80	10	35	45	14	42	56	10	28	38
0.90	10	28	38	13	35	48	10	22	32
1.00	10	22	32	11	29	40	10	18	28
90% powered main trial									
0.05	253	8880	9133	398	9149	9547	106	8511	8617
0.10	106	2292	2398	167	2400	2567	54	2154	2208
0.20	46	603	649	72	647	719	28	552	580
0.25	35	394	429	56	427	483	23	358	381
0.30	29	279	308	45	305	350	19	252	271
0.40	21	163	184	33	181	214	15	145	160
0.50	16	108	124	26	122	148	12	95	107
0.60	14	78	92	21	89	110	11	68	79
0.70	12	59	71	18	68	86	10	50	60
0.75	11	52	63	17	60	77	10	44	54
0.80	10	46	56	16	54	70	10	39	49
0.90	10	37	47	14	44	58	10	31	41
1.00	10	30	40	13	37	50	10	25	35

**Table 7.** Average power for two armed trials designed using different adjustment methods based on 10,000 simulations using 90% power, 5% Type I error rate and ‘optimal’ pilot trial sample sizes.

Standardised effect size		80% upper confidence limit	95% upper confidence limit	Non-central t-distribution
0.05	Pilot trial sample size	506	796	212
	Average power	91.25	92.51	90.52
	Percentage of trials with power above 90%	81.71	95.19	57.91
	Percentage of trials with power above 80%	100.00	100.00	99.87
0.1	Pilot trial sample size	212	334	108
	Average power	92.12	93.21	90.34
	Percentage of trials with power above 90%	82.15	95.93	60.34
	Percentage of trials with power above 80%	99.98	100.00	99.00
0.2	Pilot trial sample size	92	144	56
	Average power	93.17	94.75	90.36
	Percentage of trials with power above 90%	83.12	95.87	64.2
	Percentage of trials with power above 80%	99.74	100.00	96.15
0.5	Pilot trial sample size	32	52	24
	Average power	94.37	96.66	92.09
	Percentage of trials with power above 90%	84.19	95.60	68.90
	Percentage of trials with power above 80%	97.89	99.86	91.00
0.8	Pilot trial sample size	20	32	20
	Average power	95.37	97.60	92.10
	Percentage of trials with power above 90%	84.73	95.85	69.45
	Percentage of trials with power above 80%	95.33	99.53	89.23

**Table 8.** Estimated stepped rules of thumb for required pilot trial sample size per treatment arm when the NCT approach will be used to calculate the main trial sample size.

Standardised difference	80% powered main trial	90% powered main trial
Extra small (δ < 0.1)	50	75
Small (0.1 ≤ δ < 0.3)	20	25
Medium (0.3 ≤ δ < 0.7)	10	15
Large (δ ≥ 0.7)	10	10

### 2.3 Summary of standard methods

Setting the pilot trial sample size in order to minimise the total sample size of the pilot and the main trial together could be argued to be the most appropriate method of sample size calculation as it recognises that the pilot trial is part of a larger clinical development programme, rather than a stand-alone study. Other methods fail to recognise this point and aim to minimise both the pilot and the main trials separately which could lead to the suboptimal sample size overall.

## 3 Proposed methods of optimising the sample size across studies

Using standardised differences (δ=d/s) and pilot trial sample sizes per treatment group of 1 and upwards, we can calculate the required main trial sample sizes based on all combinations of these variables using the NCT approach through equation (4). As *n_M_* appears on both sides of equation (4), it can be solved iteratively. To calculate a starting point for the iterations we can use,

(5) $$\frac{n_{\mathrm{START}}=(r+1)s^{2}[t^{-1}(1-\beta ,k,z_{1-\alpha /2}){]}^{2}}{rd^{2}}$$

which gives a direct estimate of the sample size without iteration. Once the required main trial sample size per arm, *n_M_*, has been found, it is then added to the specified pilot trial sample size per arm, *m*, m=(k+2)/2 for a two armed design, to find the overall study sample size per arm (*N_O_*) if this design is to be used.

(6) $$N_{O}=m+n_{M}$$

For each value of δ, the pilot trial sample size per arm, $m_{\mathrm{OPT}}$, which minimises the size of the overall study, *N_O_*, can be found; this is referred to as the optimal pilot trial sample size. Therefore, if the δ to be used in the main trial is known, it is possible to calculate exactly the optimal pilot trial sample size in order to minimise the overall trial sample size. This process is depicted in Figure 1.

However, the exact δ to be used in the main trial may not be known at this early stage. Therefore, pilot trial sample size rules of thumb have been calculated based on the small, medium or large standardised effect sizes as set out by Cohen.^18^

## 4 Results

### 4.1 Optimal sample sizes

In order to calculate the minimum possible overall sample size for each standardised difference and adjustment method, the method presented in Figure 1 was used. The total sample size required for a two armed main trial for standardised differences of 0.2, 0.5 and 0.8 can be seen in Figures 2 to 4, these were calculated based on a power of 90%, Type I error rate of 5% and an allocation ratio, *r*, of 1.

It can be seen from Figures 2 to 4 that it is possible to solve the function and find the pilot trial sample size, which minimises the overall trial sample size. Table 2 shows the optimal pilot sample size, the required main trial sample size for the pilot trial and then the resulting overall trial sample size per treatment group for all adjustment methods based on a main trial power of 80%. Table 2 shows the same results but for a main trial power of 90%. The sample sizes presented in the tables are per treatment group.

The straight line on the graphs depicts a standard sample size calculation with no adjustment method applied (based on equation (1)). The points on the line show the resulting overall sample size if the rules of thumb of 24, 30 or 70 were used with no adjustment applied, the population variance is assumed to be known. The bottom dashed curve represents the NCT method as proposed by Julious and Owen.^9^ The points on the line show the resulting overall trial sample size if the rules of thumb of 24 or 30 subjects were used for the pilot trial. The middle curve is the UCL method with an 80% UCL for the variance. The points represent the rules of thumb of 20 and 40 as set out by Kieser and Wassmer^12^ as well as the 24 and 30 rules. The top dotted curve is the UCL method with a 95% UCL for the variance. The point for a pilot trial sample size of 55 has been added here, as this was the sample size recommended by Sim and Lewis^11^ to minimise the overall trial sample size. The overall trial sample sizes on the graphs are the total for a two armed trial.

The graphs in Figures 2 to 4 can be used to compare the effects of using the rules of thumb described in Table 2 to the theoretical optimal solution. For a medium standardised effect size (e.g., 0.5), the suggested rules of thumb are very close to the optimal pilot sample size. However, when the standardised effect size moves away from this value, the rules of thumb are less useful. For small standardised effect sizes (e.g., 0.2), the rules of thumb underestimate the required size of the pilot trial. For large standardised effect sizes (e.g., 0.8), the rules of thumb overestimate the number of participants required for the pilot trial. This indicates that the larger the main trial the larger the pilot trial should be in order to minimise the overall sample size; therefore; one fixed flat pilot trial sample size will not be suitable for all studies.

In relation to overall trial sample size, overestimating the pilot sample size is not as costly as underestimation in terms of over recruitment of participants as shown in Figures 2 to 4, given that the slope on the right hand side is flatter than on the left. The gradient of the slope on the right hand side of the minimum value is less than the gradient of the slope to the left side of the minimum; therefore, for the same change in pilot trial sample size – over estimation compared to underestimation – the change in overall trial sample size will be comparatively less. It can be seen that the NCT approach produces consistently lower overall trial sample sizes than any of the UCL methods.

It should be noted that for large values of standardised effect size, the suggested pilot trial sample size falls to a level, which may be considered too low to achieve the objectives of a pilot trial. This is because pilot trials are not only used to estimate the standard deviation of the outcome measure but also to assess objectives such as testing the feasibility of trial processes or predicting the likely dropout rate. We must consider these other objectives as well as more practical considerations. For the rest of this paper, a floor will be placed on the minimum pilot trial sample size per arm of 10 participants; this allows some investigation of these other objectives and is in line with the minimum sample size for an internal pilot trial sample size as recommended by Birkett and Day.^19^ Table 6 (80% powered main trial and 90% powered main trial) represents the optimal results with a floor on the lower limit of the pilot study sample size at 10 per treatment group.

It should also be noted that although the exact calculation for the NCT approach (equation 4) has been used here to gain the most accurate results, in practice using the approximation in equation 5 will result in an overall study sample size of one subject less than the exact calculation at the most.

Table 6 shows again that the NCT method produces smaller overall trial sample sizes than both the 80% and 95% UCL methods. There is, on average, no loss of power when using the NCT approach, simulations and the results can be seen in Table 7. In order to calculate the results in Table 7, a pilot trial was simulated with two treatment arms. The results were drawn from a normal distribution, the control arm with a mean of 0 and a variance of 1 and the experimental arm with a mean of the required effect size and variance of 1. Depending on the adjustment method we were looking at, the pilot trial was set to the optimal sample size for that approach and effect size. The standard deviation was estimated from the pilot trial, this was then used to calculate the sample size for the main trial (the method depending on the approach under investigation). The main trial sample size calculations were based on a Type I error rate of 5%, a Type II error rate of 10% and an allocation ratio between the treatment groups of 1. Using the same method as with the pilot trial, the main trial was then simulated based on this sample size. The results of the main trial were then tested using a *t*-test. This simulation was repeated 10,000 times for each situation. The analysis was carried out in R 3.1.2.

From the simulations, the NCT approach gives the simulated average power closest to the nominal power level. When the standardised effect size is large, the 95% UCL approach has an average power approximately 7% above the nominal value.

### 4.2 Rules of thumb revisited

In many trials, the actual value of standardised effect size to be used in the main trial may not be known before the pilot trial planning stage. This is one of the reasons that the existing rules of thumb for the pilot trial sample size, as introduced earlier in the paper, are so attractive. However, an investigator is likely to know whether the standardised difference for use in the main trial is likely to be small, medium or large within a range.

From the results presented, it would seem that any rules of thumb should be stepped – and not flat – so that the pilot is bigger for smaller standardised effect sizes and smaller for large standardised effect sizes.

Table 6 (80% powered main trial and 90% powered main trial) has been used to derive new stepped rules of thumb for the pilot trial sample size; these are presented in Table 8. These offer (per arm) sample sizes for pilot trials, which vary depending on whether the standardised effect size for the main trial is small, medium or large. An additional category of extra small has been inserted into Cohen’s classifications, which represents standardised effect sizes of 0.1 or less; this is because the results for these trials were many times larger than for standardised effect sizes of 0.2.

## 5 A worked example

A two armed parallel group randomised controlled clinical trial is being planned with a two-sided Type I error rate of 5% and a power of 90%. The primary outcome is anticipated to take a normal form. As the investigator initially was unsure about design aspects of the main trial such as the anticipated standard deviation of the outcome measure and the likely recruitment and dropout rates, a pilot trial was undertaken.

Initially a flat rule of thumb was used, and the pilot sample size was chosen to be 24 evaluable patients in total as recommended by Julious.^16^

However, suppose that *a priori* the standardised effect size for the main trial is 0.25. Using the NCT approach, the main trial sample size is estimated to be 760 participants, assuming that the pilot trial of 24 was used to design the trial. This would result in a total sample size for the pilot and main trial together of 784 participants.

As highlighted previously, if the standardised effect size to be used in the main trial is known to be 0.25 prior to the pilot trial, then based on the method presented in this paper, the optimal pilot trial sample size for a standardised difference of 0.25 is 46. If a pilot trial of 46 participants was carried out and the main trial planned based on the estimate of the standard deviation from that pilot study; then the main trial sample size based on the NCT approach would be 716. This method would result in a total overall sample size of 762 participants.

Thus, by increasing the sample size for the pilot trial, in this example nearly doubling the sample size, we have increased the precision around the standard deviation estimate. This has had the effect of reducing the total trial sample size by 22.

There are many instances where the effect size for the main trial is unlikely to be known prior to the pilot trial. However, it could be considered reasonable to have an approximate idea of the sample size of the main trial based on experience of the same population, i.e. it is anticipated that the effect size will be quite small and the sample size large. Using the stepped rules of thumb (from Table 8), the sample size would be set at 50 for the pilot trial. Consequently, the main trial sample size calculation based on a standardised effect size of 0.25 would be for 712 subjects; giving a total overall trial sample size of 762. In this example due to rounding, the total sample size is the same for the stepped rules of thumb approach and the optimal solution.

## 6 Discussion

The National Institute for Health Research Evaluation, Trials and Studies Coordinating Centre defines pilot trials in context of the planning of a future trial.^20^ Therefore, the method of minimising the sample size across trials could be thought to be the most appropriate as it treats the pilot trial as part of the whole study programme rather than a stand-alone trial. In this paper, we propose a method for estimating the sample size for a pilot trial, which uses this idea. The method introduced describes how to set the sample size of a pilot trial in order to minimise the overall trial sample size, i.e. the sample size of the pilot and main trial together, for different correction methods.

We demonstrate how the size of the pilot trial impacts on the size of the overall trial when either the UCL approach or the NCT method is used to calculate the sample size for the main trial. If the pilot trial is large, the main trial will be relatively small and if the pilot trial is small, the main trial will be relatively large. It can be seen from the results in this paper that the NCT approach provides lower overall trial sample sizes than any other method while maintaining the average power at the nominal level.

Our results show that as the sample size of a main trial increases, the size of the pilot trial should also increase. For medium effect sizes, the existing rules seem sufficient; however, as we move away from a standardised effect size of 0.5, the flat rules of thumb can over or under estimate the pilot trial sample size that would minimise the overall trial sample size. Therefore, using these flat rules of thumb would lead to more patients than theoretically required being recruited to the overall trial. This is especially seen at small standardised effect sizes.

From the results presented in this paper, we recommend using the NCT approach to set the main trial sample size in conjunction with the method presented of calculating a pilot trial sample size. Doing so will on average maintain the nominal power requirement and minimise the overall trial sample size for the pilot and the main trial together.

If simpler calculations are to be undertaken for a pilot trial sample size, we recommend using the stepped rules of thumb presented in the paper to set the pilot study sample size. However, if the standardised effect size to be used in the main trial is known, we recommend that the exact calculation be used.

In the paper, the emphasis is on estimating the sample size for pilot trials to minimize the overall sample size across both the main and pilot trial combined. However, there could be other sample size considerations such as obtaining plausible estimates of the clinical effect through precision of the confidence intervals.^21–25^ Alternatively, decision science criteria could be used to optimize the risk discharged in a clinical development prior to the start of a late phase study.^26^ In both these instances, a pilot trial is still considered in context with later definitive trials but there may already – from previous work – be good estimates of the population variance.

Finally, the methods described in the paper do have limitations. The main assumption is that the design of the main trial and the pilot trial is ostensibly the same. This may not be the case, however, which could impact on the applicability of the estimate of the standard deviation from the pilot trial.

The methods described in the paper provide a way to estimate the optimal pilot trial sample size that minimises the overall sample size for a given main trial standardised effect size. We recognise that the situation of knowing the effect size prior to the pilot study is an ideal situation and so we recommend that the stepped rules of thumb, proposed in this paper, be used and the flat rules of thumb only used as a last resort.
